# The Understanding of Scalar Implicatures in Children With Autism Spectrum Disorder: Dichotomized Responses to Violations of Informativeness

**DOI:** 10.3389/fpsyg.2018.01266

**Published:** 2018-07-23

**Authors:** Walter Schaeken, Marie Van Haeren, Valentina Bambini

**Affiliations:** ^1^Laboratory of Experimental Psychology, KU Leuven, Leuven, Belgium; ^2^Center for Neurocognition, Epistemology and theoretical Syntax (NEtS), University School for Advanced Studies IUSS Pavia, Pavia, Italy

**Keywords:** pragmatics, experimental pragmatics, autism spectrum disorder, scalar implicature, informativeness, pragmatic tolerance

## Abstract

This study investigated the understanding of underinformative sentences like “Some elephants have trunks” by children with autism spectrum disorder (ASD). The scalar term ‘some’ can be interpreted pragmatically, ‘Not all elephants have trunks,’ or logically, ‘Some and possibly all elephants have trunks.’ Literature indicates that adults with ASD show no real difficulty in interpreting scalar implicatures, i.e., they often interpret them pragmatically, as controls do. This contrasts with the traditional claim of difficulties of people with ASD in other pragmatic domains, and is more in line with the idea that pragmatic problems are not universal. The aim of this study was to: (a) gain insight in the ability of children with ASD to derive scalar implicatures, and (b) do this by assessing not only sensitivity to underinformativeness, but also different degrees of tolerance to violations of informativeness. We employed a classic statement-evaluation task, presenting optimal, logical false, and underinformative utterances. In Experiment 1, children had to express their judgment on a binary option ‘I agree’ vs. ‘I disagree.’ In Experiment 2, a ternary middle answer option ‘I agree a bit’ was also available. Sixty-six Flemish-speaking 10-year-old children were tested: 22 children with ASD, an IQ-matched group, and an age-matched group. In the binary judgment task, the ASD group gave more pragmatic answers than the other groups, which was significant in the mixed effects logistic regression analysis, although not in the non-parametric analysis. In the ternary judgment task, the children with ASD showed a dichotomized attitude toward the speaker’s meaning, by tending to either fully agree or fully disagree with underinformative statements, in contrast with TD children, who preferred the middle option. Remarkably, the IQ-matched group exhibited the same pattern of results as the ASD group. Thanks to a fine-grained measure such as the ternary judgment task, this study highlighted a neglected aspect of the pragmatic profile of ASD, whose struggle with social communication seems to affect also the domain of informativeness. We discuss the implications of the dichotomized reaction toward violations of informativeness in terms of the potential role of ASD and of cognitive and verbal abilities.

## Introduction

Being able to understand the other in a conversation does not only depend on comprehending the words used by the other. One has to understand these words in their linguistic context (integrating them with prior discourse and prior knowledge) and social context (integrating them with the environmental situation and one’s understanding of the speaker’s beliefs and intentions). Being successful in making your point to the listener depends on the same aspects. This role of the linguistic and social context in language is the domain of pragmatics (Levinson, 1983; Sperber and Wilson, 1995).

Autism Spectrum Disorder (ASD) is characterized by impairments in social communication and social interaction and by the presence of repetitive and stereotyped interests and behaviors (American Psychiatric Association, 2013). It is clear from the above characterization that pragmatic language impairments are essential to the clinical diagnosis of ASD. Pragmatic impairments are traditionally claimed to be universal in people with ASD (Volden, 2017), including difficulties in understanding idioms, metaphor, and irony (Happé, 1993; Dennis et al., 2001; Martin and McDonald, 2004; Saban-Bezalel et al., 2017; Kalandadze et al., 2018), humor (Emerich et al., 2003), ambiguous meanings (López and Leekam, 2003), and in detecting pragmatic violations in communicative interactions as well as in providing appropriate information in conversation (Capps et al., 1998; Angeleri et al., 2016). However, recent literature pointed to differences in findings, in that the extent of pragmatic disruptions in ASD seems to vary depending on the specific kind of inference at stake and on the possible mechanisms involved, either more linked to linguistic or to socio-cognitive aspects (Kissine, 2016; Andrés-Roqueta and Katsos, 2017).

In the present study, we aim to gain more insight into the ability of children with ASD to derive scalar implicatures (SIs). SIs are among the most studied types of pragmatic inferences in typical development (TD) (Papafragou and Musolino, 2003; Guasti et al., 2005), but the literature on ASD is very limited and–most interestingly–points to a domain where the performance of ASD individuals might actually be not different from that of control groups. The study of SIs in ASD might thus contribute new evidence to the debate on the pragmatic profile of this population, clarifying the extent of pragmatic difficulties and offering indications to identify the most relevant communicative domains for screening and intervention. Moreover, studying SIs in ASD might bring new insights into pragmatic theories, by pinning down aspects of the processing of scalars that might be critical in atypical development and must therefore be incorporated in theoretical models, as well as in developmental pragmatics accounts.

Scalar implicatures are based on linguistic expressions like *some*, *or*, *must*, etc. Such expressions are part of a scale organized by informativity (Horn, 1972). Examples of such scales are: <All/many/some>, <Must/should/may>, <Always/often/sometimes>. Important to understand SI is the distinction between what is said on the one hand and what is implicated on the other hand (Grice, 1975). An implicature is a component of the speaker’s meaning, which is not said and therefore should be inferred. If someone asks Kathy “Are you going to John’s dinner tonight” and Kathy answers “I have an essay to finish,” her answer meant that she is not going to John’s dinner, although that’s not what she said. In other words, Kathy did not say that she is not going, but she implied it. Combining these elements in Grice’s framework (Grice, 1975) leads to the standard account of SIs. Suppose a speaker uses a weaker term of a scale (e.g., she uses “*some* presents were beautiful”). In principle listeners assume that a speaker is trying to be cooperative and truthful (Gricean Cooperation Principle). Consequently, one more specifically expects the speaker’s contribution to be as informative as possible, and to give as much information as needed, and no more. This general principle of conversation is known as the Maxim of Quantity (the others being the Maxim of Quality, Manner, and Relevance). If therefore the speaker used the weaker term and not the stronger term (e.g., “*all* presents were beautiful”), one can infer that the stronger term does not apply, otherwise she would have used it (e.g., she meant that *not all* presents were beautiful). This line of reasoning is often referred to as a preference for the pragmatic interpretation above the logical one (Noveck, 2001).

Relevance Theory (Sperber and Wilson, 1995) builds further on this and argues that, like other inferences, also implicatures are guided by a general tradeoff between the expected cognitive gains on the one hand and the effort required to derive them on the other hand. But, like in the traditional account, SIs are context-driven inferences. Also outside classic Relevance Theory, some argue for an account where listeners access the literal interpretation of an utterance before calculating conversational implicatures (Degen and Tanenhaus, 2015). Not everyone agrees with a literal-first explanation. Levinson, for instance, argues that some of the scales, e.g., the *all/many/some*-scale, are stored in the lexicon (Levinson, 2000). Hence SIs are generated by default and will only be canceled later if they turn out not to be supported by context. Chierchia offers a grammatical account of SIs (Chierchia, 2004), whereby he agrees with Levinson that some scales are part of the lexicon. They are activated every time a weak scalar item is encountered, unless they are inhibited by certain syntactic constraints. Also other scientists argue that it is possible that an utterance immediately receives an enriched interpretation without first establishing the literal interpretation (Geurts and Pouscoulous, 2009; Geurts, 2010).

A large body of evidence in experimental pragmatics has been collected to discriminate between these alternative accounts (Noveck and Reboul, 2008). Many studies employed the classic statement-evaluation task, measuring acceptance or rejection of statements with ‘some’ in contexts where the corresponding statement with ‘all’ would also be true and hence more informative from the pragmatic point of view (Katsos et al., 2011). Conclusive evidence is not yet found. A number of findings support the view that SIs are not automatic. For instance, Experiment 4 in the study of Bott and Noveck manipulated response times (Bott and Noveck, 2004). Participants had either three seconds or were limited to only 900 ms to provide their answers. The number of SIs was 16% lower in this latter condition than in the former condition, indicating that pragmatic inferences require processing costs and are therefore not automatic. Other studies replicated or extended this finding (De Neys and Schaeken, 2007; Chevallier et al., 2008; Huang and Snedeker, 2009, 2011; Degen and Tanenhaus, 2011; Dieussaert et al., 2011; Tomlinson et al., 2013; Chemla and Bott, 2014; Heyman and Schaeken, 2015; van Tiel and Schaeken, 2017), while other studies were more critical (Feeney et al., 2004; Grodner et al., 2010).

Almost all studies on scalar implicatures are done with TD children or healthy adults. Concerning ASD, to the best of our knowledge, the literature counts only four studies on SIs. The first one showed that in general participants with ASD were as likely as the controls to derive pragmatic inferences with ‘some’ and with ‘or’ (Pijnacker et al., 2009). Interestingly, in this study the ASD group was composed of a High Functioning Autism group and an Asperger group, whereby the Asperger group did not have significant delays or difficulties in language or cognitive development. Taking these two groups into account, Pijnacker et al. (2009) observed that participants in the High Functioning Autism group gave fewer pragmatic inferences than those in the Asperger. For the ‘some’-inferences, participants in the Asperger group produced even more pragmatic inferences than the controls. The second study observed similar data with younger participants (13-years old) with the scale <and/or> (Chevallier et al., 2010). Chevallier et al. (2010) showed that ASD and TD adolescents produce similar rates of exclusive interpretation of “or” and that they responded at comparable speeds. The third study found that the adolescents with ASD (14-years old) produced a similar amount of implicatures as neurotypical adults (Hochstein et al., 2017). Nevertheless, performance was not totally the same. Although adolescents with ASD showed awareness of speakers’ mental states (when asked directly about it), they were not always considering spontaneously the speakers’ specific epistemic states when they were deriving scalar implicatures. In another study, 4–15-year-old high-functioning Mandarin-speaking children with ASD performed similarly to typical controls on underinformative sentences with ‘some’ (Su and Su, 2015). When the group was split in a younger (mean age 6.6 years, *n* = 14) and an older sample (11.7 years, *n* = 14), there was still no difference between the ASD and the TD-groups. Remarkably, however, the number of logical responses for the older groups was very low: 7 and 4% for ASD and TD groups. This is somewhat in contrast with Pijnacker et al. (2009) (24% for ASD adults) and Hochstein et al. (2017) (39% for ASD adolescents). The low number of logical responses in the Su and Su (2015) study might be caused by some minor methodological differences, but also by the language and the choice of the specific term used. In Mandarin, *youde*, *youxie*, and *yixie* can be used for a part of a whole. Some authors opt for *yixie* as the some-term (Liu and Liu, 2017), while Su and Su used *youxie*. Finally, in an ongoing study with a picture selection task, Mazzaggio et al. (2017) are observing no difference between an ASD and TD group of 7-year old children in understanding scalar underinformative sentences. Overall, these data converge to indicate that participants with ASD are capable of drawing scalar inferences. The same conclusion is supported by two other related lines of research. First, in literature not directly investigating ASD but taking into account the individual’s socio-cognitive characteristics, it is shown that the interpretation of scalar terms seems to be unaffected by the scores in the Autism-Spectrum Quotient, a questionnaire measuring the degree to which adults show autistic-like traits in their everyday behavior (Heyman and Schaeken, 2015; Antoniou et al., 2016). Second, extending from scalars to the more general domain of the Gricean Maxims, a study by Surian et al. (1996) found no differences between TD children and children with ASD and with Specific Language Impairment in detecting statements that were violating the First Quantity Maxim (‘How would you like your tea?’ ‘In a cup’), while group differences were evident for violations of the other Gricean Maxims.

Hence, most of the evidence seem to clash with the traditional view that pragmatic inference is affected in ASD (Volden, 2017) and seems more in line with the idea that pragmatic deficit are not uniform and might depend on the specific kind of mechanisms at stake (Andrés-Roqueta and Katsos, 2017). SIs could indeed be a spared domain in ASD’ communicative abilities or at least a domain where successful performance (assessed as rejection of underinformative statements) does not require a full mastery of pragmatic mechanisms. However, similar conclusions cannot be safely derived, given the limited number of studies on the topic, and the fact that SIs were tested always through the same paradigm. In this scenario, our main research question was: Is the pragmatic interpretation of scalar implicatures really unaffected by ASD?

The present study investigates the understanding of scalar implicatures in ASD, departing from previous studies in two important ways. First, our participants are 3 groups of 22 children aged 10, hence younger than in most of the previous studies (Pijnacker et al., 2009; Chevallier et al., 2010; Hochstein et al., 2017) and in a bigger sample compared with Su and Su (2015) (*N* = 14 for the young group). Second, our study assesses not only sensitivity to underinformativeness, but also different degrees of tolerance to violations of informativeness. To this purpose, although we employed the classic statement-evaluation paradigm, we also used both a binary (Experiment 1) and a ternary task (Experiment 2). A ternary statement-evaluation task includes not only an acceptance and a rejection answer option, but also a middle option. Katsos and Bishop (2011) argued that a binary task forces participants to choose between acceptance and rejection. A participant who is sensitive to the underinformativeness of a scalar expression, but also tolerant toward this pragmatic violation might opt in binary task for acceptance. Hence, under this ostensibly lack of pragmatic inferencing, true sensitivity toward informativeness might be hidden. With a ternary middle option, one can show this sensitivity for informativeness, without the potential confound of a different tolerance level. Katsos and Bishop observed that, while TD children in the binary task clearly go for acceptance responses and adults for rejection responses (Experiment 1), they both have an overwhelmingly preference for the middle option in a ternary task (Experiment 2). In other words, one can interpret the findings in the two experiments as demonstrating that in the binary task children were sensitive to underinformativeness, but refrained from categorically rejecting the underinformative statements, whereas in the ternary task true sensitivity to informativeness emerged through the possibility of showing tolerance to violations of informativeness, by choosing the middle value for underinformative statements. The ternary task was also used in a recent study on young adults with first episode psychosis (Wampers et al., 2017). With the binary task, the patient groups did not derive significantly fewer SI’s than the control group. With the ternary task, however, patients derived significantly fewer SI’s and therefore gave significantly more logical answers than the controls. In other words, the more nuanced ternary task revealed a previously not visible effect.

We thus hypothesized that in the previous research, where adults with ASD performed as controls, the forced option might have hidden differences in the tolerance level for informativeness. Hence, a more fine-grained task such as the ternary judgment might be capable not only of showing the TD children’ tolerance level, but also of revealing subtler pragmatic difficulties in the ASD group.

## Experiment 1: Binary Judgment Task

In this experiment, we examined whether children with ASD perform the same way as children without ASD on a binary judgment task. Earlier research demonstrates that children use the logical meaning of an utterance more often than adults (Noveck, 2001). Research about ASD supports the idea that people with ASD have difficulties with pragmatics (Volden, 2017), but score the same as people without ASD in different experiments requiring rejection or acceptance of underinformative scalar utterances (Pijnacker et al., 2009; Chevallier et al., 2010). Following previous studies, we expected that in this experiment children with ASD perform the same way as children without ASD. To exclude the role of cognitive capacities we used two control-groups: an IQ-matched group and an age-matched group.

### Methods

#### Participants

The participants for this experiment were 66 children between 7 and 13 years old. They all spoke fluently Dutch and came from two schools in Belgium. The children in the ASD group [*n* = 22; 17 boys, 5 girls; mean age = 10.18, range = 1.736; mean IQ (Full Scale IQ, measured with the Wechsler Intelligence Scale for Children, WISC) = 89.00, range = 13.046] came from a primary school VIBO De Ring in Turnhout. Within this group there was some comorbidity with learning disabilities and psychiatric disorders. Specifically, 3 children were diagnosed with Attention Deficit Hyperactivity Disorder, 2 with Developmental Coordination Disorder, and one with Attention Deficit Disorder; for the other 16 children in the ASD group there was no other official diagnosis or data were not available. For the IQ-matched control group, we did not use pair-wise matching, but we ensured that the average IQ was close the average IQ of the ASD group. The children in the IQ-matched control group [*n* = 22; 13 boys, 9 girls; mean age = 11.16, range = 1.974 mean IQ (Full Scale IQ, measured with the WISC) = 89.18, range = 11.652] came from the same school, following special education Type 8. Within this group, it wasn’t possible to exclude learning disorders or other disorders. Specifically, 8 children were diagnosed with Dyslexia (in 2 cases with Dyscalculia and in one case with Automatization Deficit), 4 children had other learning disorders, whereas for the remaining 10 there was no other official diagnosis. The children in the TD control group (*n* = 22; 9 boys, 13 girls; mean age = 10.23, range = 1.510) came from a primary school Klimop in Ravels-Eel. These children have no ASD and no other psychiatric disorders. This group was matched with the ASD group on the basis of age. This research has been reviewed and approved by the ethical review board SMEC of the University of Leuven. Informed consent was obtained from the participants’ parents.

#### Design

The three groups of children (ASD group, IQ-matched group, and age-matched group) had to solve 24 problems, which were constructed on the basis of two variables, that is type (scalar vs. non-scalar items) and informativeness (underinformative vs. optimal vs. logical false), in a 2^∗^3 design. Twelve of these problems contained the critical underinformative statements, testing whether the children can reject them. Half of the underinformative statements contained the word ‘some,’ creating underinformative scalar utterances. The other half were non-scalar underinformative statements. There were also 12 control problems (six for scalar and six for non-scalar expressions) with a similar structure as the critical items. Half of these problems tested whether participants could reject logically false utterances and the other half tested whether participants could accept optimal utterances, that is, utterances that are both logically true and pragmatically informative.

#### Materials

Each of the 24 problems was embedded in a story, with an animate protagonist and some objects (e.g., an elephant and trucks and busses; a dancer and red and yellow flowers). Two additional fictional characters were used: a professor and an extra-terrestrial named Frits. The professor told a story and then asked a question about the protagonist. Then the extra-terrestrial answered the question by producing the target statement, belonging to one of the six conditions. Children had to express a judgment indicating whether they agreed or not with the statement uttered by the extraterrestrial. Examples of the stories are provided in **Table 1**.

**Table 1:** Examples of stories.

Type of item	Scalar	Non-scalar
Optimal	The goat likes jumping over things. On the screen one sees fences and bushes. The professor tells that the goat jumped over three out of five fences. Next, he asks what the goat jumped over. Frits answers that the goat jumped over some of the fences.	There is a builder who likes carrying things around and there are four objects shown: a piano, a parcel, a bucket, and a ladder. The professor tells that the builder carried the bucket and the ladder. Next, he asks Frits what the builder carried. Frits answers that the builder carried the bucket and the ladder.
Underinformative	There is an elephant who likes pushing things. On the screen one sees busses and trucks. The professor tells that the elephant pushed all the trucks. Next, he asks what the elephant pushed. Frits answers that the elephant pushed some of the trucks.	There is a monkey who loves eating and four objects are shown: a banana, a cake, an orange, and a biscuit. The professor tells that the monkey ate the orange and the biscuit. Next, he asks what the monkey ate. Frits answers that the monkey ate the biscuit.
Logical false	There is a dancer who likes picking flowers. There are red flowers and yellow flowers. The professor tells that the dancer picked up three out of five red flowers. Next, he asks what the dancer picked up. Frits answers that the dancer picked up some of the yellow flowers.	The doctor likes washing his toys. On the screen one sees a bicycle, a set of drums, a car and a telephone. The professor tells that the doctor washed the car. Next, he asks what did the doctor wash. Frits answers that he washed the bicycle.


#### Procedure

Stories were presented as a slideshow (constructed with Microsoft Power Point software) on a computer. Visual stimuli were accompanied by auditory stimuli, with the prerecorded voices of the professor and of the extraterrestrial. The experiment started with Frits, an extra-terrestrial who introduced himself. He explained he was visiting the Earth and wanted to learn Dutch better. He proposed the child to help him with it. Next, a professor appeared on the screen and introduced the task.

**FIGURE 1:**
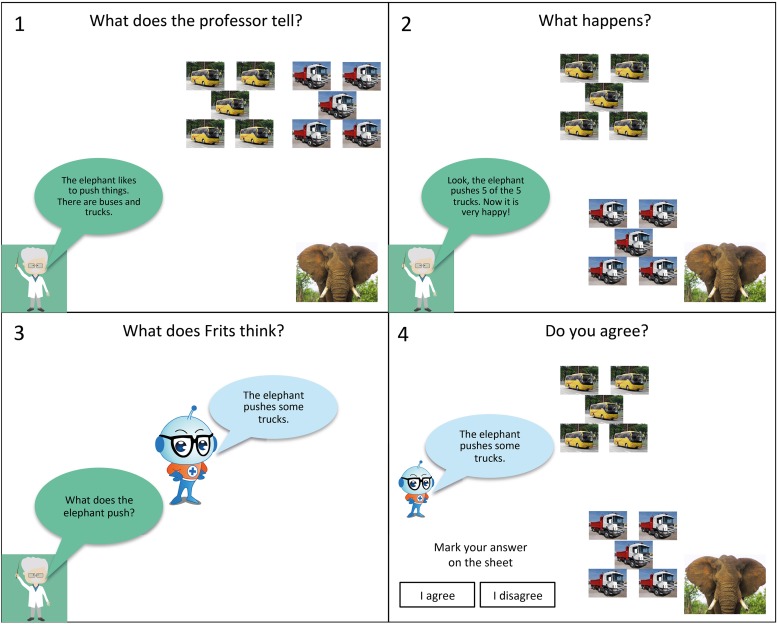
Example of presentation procedure for a story in the scalar underinformative condition in Experiment 1.

Each story was introduced by a screen with the extraterrestrial saying that the story was about to begin. Then each story was displayed in four consecutive screens. In the first screen (with the title ‘What does the professor tell?), the professor appeared, and he introduced the protagonist of the story and some objects. The second screen (with the title ‘What happens?) showed an action of the protagonist with some of the objects, while the professor described what was happening. In the next screen (with the title ‘What does Frits think?’), the professor asked Frits what the protagonist did and then Frits gave his answer. The last screen of each story (with the title ‘Do you agree or not?’) showed a summary of the story and Frits’ answer. The children were asked whether they agreed with this answer. Children had their own answer sheet with 24 items. After each story they had to mark one out of two options: ‘I agree’ or ‘I disagree.’ The children were not asked to explain why they chose a specific answer. When the participants hesitated about the answer, they were encouraged to choose between the response options. The procedure is illustrated in **Figure 1** (objects taken from Pixabay, a community of creatives, sharing copyright free images and videos. All contents are released under Creative Commons CC0).

The task took about 20 min to complete. The children from the ASD group and the IQ-matched group were picked up from their class and were taken to a quiet room in the school building. They completed the test in pairs of two, except for two children who completed the test individually. For this experiment, a laptop was used to present the slideshow. The children from the age-matched group completed the experiment classically, with the slideshow presented on a digi-board. All children were all given their answer sheet before the experiment began.

Each answer was scored as either logical correct or logical incorrect. For optimal sentences, logical correct means a choice for ‘I agree’; for underinformative sentences logical correct again means ‘I agree’ and for logical false sentences it means ‘I disagree’.

#### Data Analysis

We performed two analyses on the data. Given the binary nature of the dependent variable, we performed a mixed effects logistic regression (Baayen et al., 2008; Jaeger, 2008; Bates et al., 2015). The model fitting procedure was implemented in R using the glmer() function from the lme4 package (Bates et al., 2015). The dependent variable was the accuracy score (logical correct or logical incorrect). The independent variables were Group (with the levels ASD group, IQ-matched group, and age-matched group), Informativeness-Type (with the levels underinformative, optimal, and logical false items), and Scalar-Type (with the levels Scalar and Non-Scalar items). All models included random intercepts for participants and items. We started with the most complex fixed effects structure, including the three-way interaction between Group, Informativeness-Type, and Scalar-Type, as well as all two-way interactions and main effects. We then used backward elimination, which involved simplifying the model by removing interactions that did not contribute significantly as evaluated via a likelihood ratio test (*p* < 0.05). We verified that model fitting through the Akaike information criterion (AIC) would have lead to the same model selection.

To facilitate the comparison with Katsos and Bishop (2011) and to increase transparency through a multiverse analysis (Steegen et al., 2016), we also performed a non-parametric analysis similar to the one in Katsos and Bishop’s original study. This analysis is in line with the fact that our data (as their data) were not normally distributed and with the binary nature of the dependent variable. Overall between-subject effects were checked by means of Kruskal–Wallis one-way analysis of variance, a non-parametric method for comparing two or more independent samples. Between-subject effects were further analyzed by means of a Mann–Whitney *U* test, whereas within-subject effects were analyzed by means of a Wilcoxon Signed Ranks tests.

### Results

#### Mixed Effects Logistic Regression Analysis

**Table 2** presents the answers of the children. The final model included a two-way interaction between Group and Informativeness-Type and between Informativeness-Type and Scalar-Type, in addition to all three main effects. For a complete description of the final model, see **Table 3**. The significant interactions were further analyzed by pairwise contrasts, using Bonferroni corrected lsmeans (). This revealed two significant differences for the interaction between Group and Informativeness-Type, that is, the ASD group gave more pragmatic answers on the underinformative items than the age-matched group (*Z* = -6.155, *p* < 0.0001) and the IQ-matched group (*Z* = -4.267, *p* = 0.0001). The significant interaction between Informativeness-Type and Scalar-Type was due to the difference between the scalar and non-scalar items on the underinformative items (*Z* = 3.480, *p* = 0.0005).

**Table 2:** Experiment 1: percentage of *I agree* responses for the three item types.

		ASD group		Age-matched group		IQ-matched group	
				
Type of item	Type of response	Scalar	Non-scalar	Scalar	Non-scalar	Scalar	Non-scalar
Optimal	*I agree* (=logical and pragmatic)	96.97%	96.97%	98.48%	100%	93.94%	95.45%
Underinformative	*I agree* (=logical, not pragmatic)	46.97%	63.64%	72.73%	73.48%	72.73%	88.64%
Logical false	*I agree* (=false)	0%	4.55%	1.52%	4.55%	6.06%	6.06%


**Table 3:** A complete description of the final model for Experiment 1: Group^∗^Informativeness Type + Informativeness Type ^∗^ Scalar Type + (1| Participant) + (1| Item).

**Estimators of the relative quality of the statistical model:**								
**AIC**	**BIC**	**logLik**	**Deviance**	**df.resid**				
830.7	905.9	-401.4	802.7	1570				
**Scaled residuals:**								
**Minimum**		**1Q**		**Median**			**3Q**	**Maximum**
-22.5187		0.0108		0.0830			0.2087	2.6028
**Random effects:**								
**Groups**		**Name**		**Variance**		**Standard deviation**		
Participant		(Intercept)		8.821		2.97		
Item		(Intercept)		0.000		0.00		
Number of obs: 1584, groups: Participant, 66; Item, 24								
**Fixed effects^∗^:**								
				**Estimate**		**Standard error**	***z*-value**	**Pr(>|z|)**
(Intercept)				5.4977		0.9664	5.689	0.00000001280
Group_IQ				-0.7561		1.2032	-0.628	0.529752
Group_AGE				4.1211		1.6495	2.498	0.012476
INFO TYPE_UI				-4.2243		0.7294	-5.792	0.00000000697
INFO TYPE_FA				-0.2255		0.9069	-0.249	0.803651
SCALAR TYPE_Sc				-0.3854		0.6258	-0.616	0.537937
Group_IQ:INFO TYPE_UI				2.8435		0.7770	3.659	0.000253
Group_AGE:INFO TYPE_UI				-1.8772		1.3231	-1.419	0.155957
Group_IQ:INFO TYPE_FA				-0.4885		0.9870	-0.495	0.620649
Group_AGE:INFO TYPE_FA				-1.8712		1.4173	-1.320	0.186756
INFO TYPE_UI:SCALAR TYPE_Sc				-0.7311		0.6684	-1.094	0.274043
INFO TYPE_FA:SCALAR TYPE_Sc				1.1983		0.8600	1.393	0.163507


*^∗^The fixed effects are Group (with the levels ASD group, IQ-matched group, and age-matched group), Informativeness-Type [with the levels underinformative (UI), logical false items (FA) and optimal], and Scalar-Type [with the levels Scalar items (Sc) and Non-Scalar items].*

#### Non-parametric Analyses

Concerning the differences between the three groups, Kruskal–Wallis tests show that there is no significant main effect of group in agreeing with the optimal condition (scalar items: χ = 1.666, *p* = 0.581; non-scalar items: χ = 3.527, *p* = 0.312) and in disagreeing with the logical false condition (scalar items: χ = 3.715, *p* = 0.242; non-scalar items: χ = 0.005 *p* = 1.000). Furthermore, the null hypothesis that there is no difference between the groups in answering to the underinformative items could not be rejected either (scalar items: χ = 5.267, *p* = 0.072; non-scalar items: χ = 4.386, *p* = 0.111).

Concerning the comparisons between types of items within the groups, pairwise comparisons by using Wilcoxon Signed Ranks tests show that the children gave significantly more logical correct answers in the logical false condition and in the optimal condition compared with the underinformative condition, and this both for the scalar items (optimal vs. underinformative, *W* = -2.333, *p* = 0.031; logical false vs. underinformative, *W* = -2.349, *p* = 0.031) and the non-scalar items (optimal vs. underinformative, *W* = -2.456, *p* = 0.016; logical false vs. underinformative, *W* = -2.388, *p* = 0.016). There was no significant difference between scalar and non-scalar items (*W* < 0.0001, *p* = 0.625).

A similar pattern was observed for the ASD group. These children gave more logical correct answers in the logical false condition and in the optimal condition compared with the underinformative condition, and this both for the scalar (optimal vs. underinformative, *W* = -3.247, *p* < 0.0001; logical false vs. underinformative, *W* = -3.352, *p* < 0.0001) and the non-scalar items (optimal vs. underinformative, *W* = -2.931, *p* = 0.001; logical false vs. underinformative, *W* = -3.310, *p* < 0.0001). Also in this group there was no significant difference between scalar and non-scalar items (*W* = -1.604, *p* = 0.114).

The children in the IQ-matched group had a slightly different pattern. They gave more logical correct answers in the logical false condition and in the optimal condition compared with the underinformative condition for scalar items (optimal vs. underinformative *W* = -2.086, *p* = 0.037; logical false vs. underinformative *W* = -2.239, *p* = 0.026). Conversely, there were no significant differences with respect to the non-scalar items (optimal vs. underinformative, *W* = -1.691, *p* = 0.086; logical false vs. underinformative, *W* = -0.966, *p* = 0.344). The difference between scalar and non-scalar items was significant (*W* = -2.161, *p* = 0.029).

### Discussion of Experiment 1

In this experiment, we expected that children with ASD would perform the same way as children without ASD. The two analyses confirm this hypothesis for the optimal items and the logical false items. However, inspection of the data revealed that individuals with ASD gave fewer ‘I agree’ answers (i.e., fewer logical answers and thus more pragmatic answers) than the other groups, which was significant in the mixed effects logistic regression analysis, although not in the non-parametric analysis. This finding is in line with the finding of Pijnacker et al. (2009). They observed that the Asperger group was even better at deriving scalar implicatures than the control group. Hence our finding offers further evidence to the idea that, under a binary task, ASD individuals are not less and maybe even more competent than controls in informativeness.

The regression analysis also revealed a significant difference between the scalar and the non-scalar underinformative items, the latter being easier than the former. At first sight, this is in contrast with research showing that non-scalar or *ad hoc* implicatures are easier for young children (Papafragou and Tantalou, 2004; Stiller et al., 2015; Horowitz et al., 2017). In the Katsos and Bishop study, on which ours is based, children’s performance did not significantly differ between the scalar and non-scalar expressions in the underinformative condition. We will discuss this issue further in the Section “General Discussion.”

Previous research already showed that the answer alternatives are crucial in experiments with children, because binary answer alternatives can cloud sensitivity to informativeness (Katsos and Bishop, 2011). Therefore, we set up a second experiment to investigate whether the use of different answer alternatives would have influence on the results of children with ASD. This reasoning also applies to cognitive differences, because the IQ of the children also made no differences in this binary judgment task.

## Experiment 2: Ternary Judgment Task

In their study, Katsos and Bishop (2011) did a second experiment by presenting a ternary answer alternatives, and proved that children are competent with informativeness but also tolerant to violations of informativeness. Specifically, when a middle option is available, children tend to prefer it to the extremes, thus showing that they are sensitive to informativeness but also tolerant to its violation. In our research, we did a second experiment to investigate whether the same underlying mechanism can be found in children with ASD. We investigated whether the children with ASD were as tolerant to informativeness as children without ASD. This experiment could uncover subtle difficulties that people with ASD might have with the pragmatic dimension of informativeness, which might be disguised by the forced option in the binary task.

### Method

#### Participants

The procedure and the participants of this experiment were the same as in Experiment 1. Experiment 2 followed Experiment 1 after a short break.

#### Design, Materials, and Procedure

In this experiment we used the same design, materials, and procedure as in Experiment 1. The difference with the previous experiment was in the fourth screen, where the alternatives are displayed, and in the answer sheet. The children didn’t have to choose between ‘I do agree’ and ‘I disagree.’ In this experiment they had three answer options, namely: ‘I totally agree,’ ‘I agree a bit,’ and ‘I totally disagree.’ Following previous work (Katsos and Bishop, 2011), these options were represented with strawberries: a little strawberry on the left of the row (‘I totally disagree’), a big strawberry in the middle of the row (‘I agree a bit’), and a huge strawberry on the right of the row (‘I totally agree’). On the top of the answer sheet there was a legend with the strawberries and their explanation.

#### Data Analysis

As for Experiment 1, we performed two analyses. First, we performed a mixed effect ordinal regression analysis. The model fitting procedure was implemented in R using the clmm() function from the ordinal package (Christensen, 2015). The dependent variable was an agreement score (0, 1, 2: I totally disagree, I agree a bit, I totally agree, respectively). The independent variables were Group [(with the levels ASD group, IQ-matched group, and age-matched group), Informativeness]-Type (with the levels underinformative, optimal, and logical false items), and Scalar-Type (with the levels Scalar and Non-Scalar items). All models included random intercepts for participants and items. We used the same backward elimination procedure as in Experiment 1.

Second, because the data were not normally distributed, given the binary nature of the dependent variable, and to enable a straightforward comparison with previous research (Katsos and Bishop, 2011), we also did a non-parametric analyses. Overall between-subject effects were checked by means of Kruskal–Wallis one-way analysis of variance, a non-parametric method for comparing two or more independent samples. Overall within-subject effects were checked by means of a Friedmann ANOVA, a non-parametric method for comparing two or more dependent samples. Between-subject effects were further analyzed by means of a Mann–Whitney *U* test, whereas within-subject effects were analyzed by means of a Wilcoxon Signed Ranks tests.

### Results

#### Mixed Effect Ordinal Regression Analysis

**Table 4** presents the answers of the children. The final model included a two-way interaction between Group and Informativeness-Type and between Group and Scalar-Type, in addition to all three main effects. For a complete description of the final model, see **Table 5**. The significant interactions were further analyzed by pairwise contrasts, using lsmeans (). This revealed – for the interaction between Group and Informativeness-Type – three significant differences. On the underinformative items, the age-matched group differed from the ASD group (*Z* = -2.498, *p* = 0.0374) and from the IQ-matched group (*Z* = -3.044, *p* = 0.007). On the optimal items, the age-matched group differed from the IQ-matched group (*Z* = -2.611, *p* = 0.0271). The significant interaction between Group and Scalar-Type was due to a difference between the scalar and non-scalar items both in the ASD group (*Z* = 3.065, *p* = 0.0022) and in the IQ-matched group (*Z* = 2.621, *p* = 0.0088).

**Table 4:** Experiment 2: percentage of each response type for the three item types.

		ASD group		Age-matched group		IQ-matched group	
				
Type of item	Type of response	Scalar	Non-scalar	Scalar	Non-scalar	Scalar	Non-scalar
Optimal	*I totally disagree*	0%	1.52%	0%	1.52%	3.03%	1.52%
	*I agree a bit*	1.52%	0%	6.06%	1.52%	6.06%	4.55%
	*I totally agree*	98.48%	98.48%	93.93%	96.97%	90.91%	93.94%
Underinformative	*I totally disagree*	40.15%	16.67%	3.03%	4.55%	29.55%	3.79%
	*I agree a bit*	47.73%	62.88%	87.88%	90.15%	52.27%	74.24%
	*I totally agree*	12.12%	20.45%	9.10%	5.30%	18.18%	21.97%
Logical false	*I totally disagree*	98.48%	80.30%	83.33%	62.12%	96.97%	78.79%
	*I agree a bit*	0%	16.67%	16.67%	36.36%	1.52%	21.21%
	*I totally agree*	1.52%	3.03%	0%	1.52%	1.52%	0%


**Table 5:** A complete description of the final model for Experiment 2: Group ^∗^ Scalar Type + Group^∗^Informativeness Type + (1|Participant) + (1|Item).

**Estimators of the relative quality of the statistical model:**						
**Link**	**Thresholdnobs**	**LogLik**	**AIC**	**Niter**	**Max.grad**	**cond.H**
logit flexible	1584	-809.01	1648.01	1146(4720)	1.97e-04	1.9e+02
**Random effects:**						
**Groups**	**Name**	**Variance**	**Standard deviation**			
Participant	(Intercept)	1.0114	1.0057			
Item	(Intercept)	0.9054	0.9515			
Number of groups: Participant 66, Item 24						
**Fixed effects^∗^:**						
		**Estimate**	**Standard error**	***z*-value**	**Pr(>|z|)**	
Group_ASD		-1.51679	0.58914	-2.575	0.010036	
Group_IQ		-1.29266	0.56247	-2.298	0.021552	
Scalar Type_Sc		0.01683	0.46627	0.036	0.971209	
Info Type_OP		9.328260635	0.80635	11.569	<0.0000000000000002	
Info Type_UI		3.70847	0.56769	6.533	0.0000000000647	
Group_ASD: Scalar Type_Sc		-1.55554	0.36108	-4.308	0.0000164710512	
Group_IQ:Scalar Type_Sc		-1.28026	0.34389	-3.723	0.000197	
Group_ASD:INFO TYPE_OP		4.15855	1.06737	3.896	0.0000977631243	
Group_IQ:INFO TYPE_OP		1.47938	0.75806	1.952	0.050993	
Group_ASD:NFO TYPE_UI		1.56495	0.54056	2.895	0.003791	
Group_IQ:INFO TYPE_UI		1.90608	0.50694	3.760	0.000170	


*^∗^The fixed effects are Group (with the levels ASD group, IQ-matched group, and age-matched group), Informativeness-Type [with the levels optimal (OP), underinformative (UI), logical false items], and Scalar-Type [with the levels Scalar items (Sc) and Non-Scalar items].*

#### Non-parametric Analyses

Concerning the differences between the three groups, three Kruskal–Wallis tests were performed. First, a Kruskal–Wallis test revealed no differences for the optimal items, and this for both the scalar and for the non-scalar items.

Second, for the logical false items, a Kruskal–Wallis test revealed a significant main effect of group in the scalar items for the answer-possibility ‘I agree a bit’ (χ = 7.829, *p* = 0.015). A follow-up Mann–Whitney *U* test showed that the effect was due to a significant difference between the ASD group and the age-matched group (*U* = 187.000, *p* = 0.048). A Kruskal–Wallis test revealed a significant main effect of group in the non-scalar items for the answer-possibility ‘I totally disagree’ (χ = 13.073, *p* = 0.001). A follow-up Mann–Whitney *U* test showed that the effect was due to significant difference between the ASD group and the age-matched group (*U* = 128.000, *p* = 0.001) and between the age-matched group and the IQ-matched group (*U* = 137.000, *p* = 0.003). A Kruskal–Wallis test also revealed a significant main effect of group in the non-scalar items for the answer-possibility ‘I agree a bit’ (χ = 14.196, *p* = 0.001). A follow-up Mann–Whitney *U* test showed that the effect was due to significant difference between the ASD group and the age-matched group (*U* = 115.500, *p* < 0.001) and between the age-matched group and the IQ-matched group (*U* = 144.000, *p* = 0.005).

Third, and most importantly, for the underinformative items, a Kruskal–Wallis test revealed a significant difference for the answer-possibilities ‘I totally disagree’ for both the scalar and the non-scalar items. For the scalar items (χ = 11.455, *p* = 0.003), a follow-up Mann–Whitney U showed that the effect was due to a significant difference between the ASD group and the age-matched group (*U* = 134.000, *p* = 0.001) and between the IQ-group and the age-matched group (*U* = 138.000, *p* = 0.002). For the non-scalar items (χ = 8.730 *p* = 0.013) a follow-up Mann–Whitney *U* showed that the effect was due to significant difference between the ASD group and the age-matched group (*U* = 178.000, *p* = 0.046) and between the ASD group and the IQ-matched group *U* = 176.500, *p* = 0.030). A Kruskal–Wallis test also revealed a significant difference for the answer-alternative ‘I agree a bit’ for both types of items. For the scalar items (χ = 10.190, *p* = 0.006) a follow-up Mann–Whitney *U* showed that the effect was due to significant difference between the ASD group and the age-matched group (*U* = 132.500, *p* = 0.006) and between the age-matched group and the IQ-matched group (*U* = 125.000, *p* = 0.004). For the non-scalar items (χ = 7.231, *p* = 0.025) a follow-up Mann–Whitney *U* showed that the effect was due to significant difference between the ASD group and the age-matched group (*U* = 138.000, *p* = 0.009).

Concerning the comparisons between types of items within the groups, three Friedman’s ANOVA tests were performed. First, in the age-matched group a Friedman’s ANOVA showed similar results as Katsos and Bishop (2011). There were significant differences in the responses to every type of utterance. This was the case for scalar items (optimal χ = 41.943, *p* < 0.001; logical false χ = 26.986, *p* < 0.001; underinformative χ = 35.553, *p* < 0.0001) and non-scalar items (optimal χ = 42.706, *p* < 0.0001; logical false χ = 36.169, *p* < 0.0001; underinformative χ = 35.370, *p* < 0.0001). Wilcoxon Signed Rank tests showed that the ‘I agree a bit’-answers were more present in the underinformative conditions (scalar *W* = -4.168, *p* < 0.0001 and *W* = -3.967, *p* < 0.0001; non-scalar *W* = -4.148, *p* < 0.0001 and *W* = -4.116, *p* < 0.0001); the ‘I totally agree’-answers were more present in the optimal conditions (scalar *W* = -4.400, *p* < 0.0001 and *W* = -4.169, *p* < 0.0001; non-scalar *W* = -4.456, *p* < 0.0001 and *W* = -4.246, *p* < 0.0001); and the ‘I totally disagree’-answers were more present in the logical false conditions (scalar *W* = -4.233, *p* < 0.0001 and *W* = -4.127, *p* < 0.0001; non-scalar *W* = -4.307, *p* < 0.0001 and *W* = -4.291, *p* < 0.0001).

Second, and similarly to the TD group, in the ASD group a Friedman’s ANOVA showed significant differences in the response to every type of utterance for the non-scalar items (optimal χ = 43.373, *p* < 0.0001; logical false χ = 38.810, *p* < 0.0001; underinformative χ = 11.760, *p* = 0.002). For the scalar items, however, significant differences can only be found in the optimal condition (χ = 43.373, *p* < 0.0001) and in the logical false condition (χ = 43.373, *p* < 0.0001). Wilcoxon Signed Rank tests showed that the ‘I agree a bit’-answers were more present in the non-scalar underinformative condition (*W* = -3.572, *p* < 0.0001 and *W* = -3.599, *p* < 0.0001); the ‘I totally agree’-answers were more present in the optimal conditions (scalar *W* = -4.523, *p* < 0.0001 and *W* = -4.197, *p* < 0.0001; non-scalar *W* = -4.456, *p* < 0.0001 and *W* = -4.024, *p* < 0.0001); and the ‘I totally disagree’-answers were more present in the logical false conditions (scalar *W* = -4.600, *p* < 0.0001 and *W* = -3.710, *p* < 0.0001; non-scalar *W* = -4.261, *p* < 0.0001 and *W* = -3.938, *p* < 0.0001).

Third, in the IQ-matched group a Friedman’s ANOVA showed a similar pattern as the ASD group, that is significant differences in the responses to every type of utterance for the non-scalar items (optimal χ = 41.600, *p* < 0.0001; logical false χ = 41.200, *p* < 0.0001; underinformative χ = 26.605, *p* < 0.0001) and to two types for the scalar items (optimal χ = 37.380, *p* < 0.0001; logical false χ = 42.706, *p* < 0.0001). Wilcoxon Signed Rank tests in the IQ-matched group show that the ‘I agree a bit’-answers were more present in the non-scalar underinformative conditions (non-scalar *W* = -4.052, *p* < 0.0001 and *W* = -4.106, *p* < 0.0001); the ‘I totally agree’-answers were more present in the optimal conditions (scalar *W* = -4.310, *p* < 0.0001 and *W* = -3.983, *p* < 0.0001; non-scalar *W* = -4.400, *p* < 0.0001 and *W* = -4.028, *p* < 0.0001); and the ‘I totally disagree’-answers were more present in the logical false conditions (scalar *W* = -4.455, *p* < 0.0001 and *W* = -3.872, *p* < 0.0001; non-scalar *W* = -4.284, *p* < 0.0001 and *W* = -4.253, *p* < 0.0001).

## General Discussion

This study aimed at investigating the pragmatic competence of children with ASD, focusing on an aspect that has remained poorly explored in the literature, namely their ability to deal with scalar implicatures and more generally with informativeness. The main interest of this study stems from the fact that there is evidence that scalars are a domain where pragmatic abilities are unaffected in ASD, and we aimed at questioning this idea with a refined experimental approach, to contribute to the description of the pragmatic profile of this population. Experiment 1 used the classic statement-evaluation task with a binary option, where subjects are required to accept or reject a statement belonging to the optimal, logical false or underinformative condition. The two analyses showed that there are no differences on the optimal and the logically false items between the ASD group and the other two groups, i.e., the age-matched and the IQ-matched. Focusing on the underinformative condition, the mixed effects logistic regression analysis additionally shows that the ASD children gave significant more pragmatic responses on the underinformative statements compared with the two other groups. This observation is in agreement with Pijnacker et al. (2009), who observed that their Asperger group was even better at deriving scalar implicatures. This difference on the underinformative statements between the ASD-children and the two other groups was, however, not significant with the non-parametric analysis, which is in line with some other evidence (Su and Su, 2015). Hence under a binary task, ASD individuals are not less and maybe even more competent than controls in informativeness. Surprisingly, in the regression analysis, there was a significant interaction between Scalar-Type and Informativeness-Type, i.e., the scalar underinformative items were easier than the non-scalar items, which contrasts with research showing the opposite pattern for young children (Papafragou and Tantalou, 2004; Stiller et al., 2015; Horowitz et al., 2017).

The key finding of the current study is the outcome of Experiment 2, where the statement verification task was used with a ternary option, which allows individuals not merely to dichotomize their answers into logical vs. pragmatic, but also to show tolerance toward violation of informativeness. Here we observed that the three groups differed significantly. Most importantly, the ASD group gave fewer ‘I agree a bit’ and more ‘I totally disagree’ answers for the underinformative items than the age-matched group. Children in the IQ-matched group performed similarly to the ASD group on the scalar underinformative items. In other words, the children in the two clinical groups split their answers between the two extreme options more than TD children, who in contrast clearly opted for the middle option ‘I agree a bit.’ Furthermore, the regression analysis of Experiment 2 revealed a significant interaction between Group and Scalar-Type. The ASD and the IQ group went more for the “I agree a bit”-answers with the non-scalar items than with the scalar items in both the logical false (which is inappropriate) and the underinformative conditions (which is appropriate). Together with the significant interaction between Scalar-Type and Informativeness-Type in Experiment 1, this suggests that inferences with non-scalar items are not similar to inferences with scalar items. This issue will be discussed in depth at the end of this general discussion.

The first consideration stemming from these data concerns the ternary task. As observed in the study of Katsos and Bishop (2011), the introduction of a middle option offers a more fine-grained measure compared with the binary task: It allows observing not just competence with informativeness, but also tolerance to underinformativeness. This proved to be crucial in developmental studies, to highlight an aspect of the children’ pragmatic competence that is hidden under the binary task where children refrain to reject underinformative statement. The ASD group does not show the pragmatic tolerance observed in the same age TD group, and continues to largely split the answers between the two extremes. We therefore showed that a finer grained measure such as the binary task is crucial for ASD research too. Children’s pragmatic sensitivity seems to be extremely task-dependent (Grigoroglou and Papafragou, 2017), and so do pragmatic difficulties. Interestingly, also Wampers et al. (2017) observed a more convincing difference between patients with first episode psychosis and controls in the ternary judgment task compared with the binary, reporting significantly more logical answers and fewer SIs for patients. These findings demonstrate the importance of this seldom used paradigm, currently employed only in very few studies (Katsos and Bishop, 2011; Pipijin and Schaeken, 2012; Wampers et al., 2017), for a detailed charting of pragmatic abilities with underinformative statements, both for TD children and clinical populations.

There is, however, one important methodological caveat linked to the ternary task as used in our study. In our setting, all children first did Experiment 1 (binary) and next Experiment 2 (ternary); therefore they had to switch between rules (first using “I agree-I disagree,” next “I totally disagree-I agree a bit-I totally agree”). This might have caused a potential confound, given the well-documented difficulties that children with ASD have with changing rules and flexibility in general (Zelazo et al., 2002; Zelazo, 2006). However, we had good reasons to use this order and we believe that this is not compromising our study. First, we opted for this procedure because this enabled us to replicate Katsos and Bishop (2011), who also used this fixed order. Our data on TD children were indeed completely in line with their results, ensuring us that Dutch-speaking children are indeed both sensitive to and tolerant of violations of informativeness with scalar and non-scalar expressions. Second, looking at answers in Experiment 2, the ASD children seem to show no problem whatsoever with the new rule, given the fact that they provided the expected responses in all conditions. Specifically, they chose overwhelmingly the “I totally agree”- and the “I totally disagree”-answers for the optimal and logical conditions, respectively; for the critical underinformative items, logical and pragmatic answers were also overwhelmingly given (84% of the answers are “I totally disagree” or “I agree a bit”), again indicating that the rule-switching was not causing problems. Finally, the absence of a rule-switching setback might not be so surprising if one compares our rule-switching with the one in the dimensional change card sort (DCCS), often used to document the difficulties with flexibility. In the DCCS, the participants have to sort a series of bivalent cards. First, they have to sort them according to one dimension (e.g., color), next to another (e.g., shape). Most 5-year old children perform well on this task; however, children with ASD perform worse (Zelazo et al., 2002; Zelazo, 2006). In contrast with the DCCS, the switching from Experiment 1 to Experiment 2 did not require to use an alternate dimension. Our children had still to use the same dimension, although it was only a bit more nuanced (i.e., with a middle point) in Experiment 2. Given all this, we believe that our results are not affected by the potential confound of flexibility difficulties. Nevertheless, this issue should be investigated systematically in follow-up research.

In the next part of the Discussion, we focus on the contribution of our study to the description of the global pragmatic profile of ASD, taking into account that the dichotomized answer pattern is observed also in the IQ-matched group, and discussing the potential role of intellectual, verbal, and theory of mind abilities.

First, our study has important implications for the research on communicative abilities in ASD. The pragmatic profile of ASD is classically sketched as a pervasive difficulty with social communication, surfacing in a large range of contexts, from conversation to the understanding of implicit meanings. Pragmatic impairments in ASD are claimed to be universal (Volden, 2017) and to be evident at all developmental stages, even in highly verbal adults with ASD (Groen et al., 2008). However, recent literature evidenced that the deficit is not uniform, as there are different kinds of pragmatic inferences, which might be spared and might in turn be linked to individual differences in verbal and ToM skills (Kissine, 2016; Andrés-Roqueta and Katsos, 2017). For instance, children with high functioning ASD are able to extract the speakers’ state (e.g., physical, emotional, and social) from their prosodic cues, although they might have subtle difficulties when cognitive loads are higher, i.e., in most challenging contexts such as real-life social situations where multiple cues need to be rapidly integrated (Chevallier et al., 2011). Also, there is evidence that children with ASD are able to interpret indirect requests when tested in naturalistic settings (Kissine et al., 2015).

Scalars are a crucial domain in this debate, as some studies, although limited in number, pointed to a preserved capacity in ASD (Pijnacker et al., 2009; Chevallier et al., 2010; Su and Su, 2015). Our data shed new light on this issue, suggesting that pragmatic challenges might indeed extend to the domain of informativeness and scalar implicatures, although this difficulty might be not visible under a standard binary task. More specifically, in our study the pragmatic challenges of ASD take the shape of a dichotomized response pattern for scalars in a ternary task. This pattern might be indicative of a difficulty at the inferential level, of genuine Gricean pragmatic type. A scalar expression requires the ability to infer an implicit content, whereby the listener has to make a series of inferences. First, the children in our experiments had to infer that Frits, the speaker, could have used the stronger term. So, they had to be able to come up with the stronger alternative themselves. Second, they had to decide what the choice of the weaker term implied. Did it mean that the speaker made a clear mistake, or that according to the speaker the stronger term did not hold or was it only a sentence that was “a bit unlucky formulated”? It might be that the children with ASD went more often than the TD children for the first inference than the last and rejected Frits’s statement, because they have difficulties in envisaging the possibility of less appropriate, but not completely wrong language use. Hence, based on our findings, in describing the pragmatic profile of ASD, researcher should include difficulties in sensitivity to informativeness.

One exception to most studies is a recent study from this research topic (Pastor-Cerezuela et al., 2018), which evaluates the comprehension of generalized conversational implicatures (GCI) in children with and without ASD using a purely verbal test that is based on the Levinson model of implicatures (Levinson, 2000). This study observed that the ASD group performed worse than the controls for each implicature type, so also for the implicature type related to the Q (or scalar) heuristic. Because there were only five items testing the Q-heuristic, from which there was only one underinformative item with ‘some,’ one has to be careful with the interpretation, but it is striking that also this task did not have a straightforward binary answer format. Indeed, for the *some*-statement “Some guests came to Maria’s party,” the response options were: (a) “All the people Maria invited came”; (b) “Not all the guests Maria expected came”; and (c) “Exactly three guests came.” These response options are not the same as the options in our ternary task, but offering more than two response options might be a crucial aspect, which needs to be investigated further in the future.

A critical issue when claiming a communicative impairment in ASD is the consideration of intellectual and verbal abilities. Indeed, our study observed no significant differences between the ASD and the IQ-matched groups in the ternary task. This result points to the fact that global intellectual abilities play a role in pragmatics, specifically in inferring from the Quantity maxims, and that ASD abilities with scalars are commensurate to their intellectual abilities. The measure of intelligence employed here (WISC) included both performance and verbal aspects. Although we cannot disentangle which of the two components played a major role, it is likely that both components impacted on scalar processing. Concerning verbal intelligence, its impact is well known for other pragmatic domains. A recent meta-analysis showed that, across studies, ASD children seem to have poorer comprehension of figurative language compared with TD peers; however, when matched for verbal abilities, the two groups do not differ significantly, and thus the deficit seems to be neither universal nor unique to individuals with ASD (Kalandadze et al., 2018). Although scalar expressions are different from figurative language, both are considered to be linked to linguistic-based inferences (Andrés-Roqueta and Katsos, 2017). To solve a scalar inference, children need to draw on semantic knowledge of the meaning of ‘some’ and ‘all’ and on the fact that the latter is more informative than the former. Although the measure we used is considered by some not ideal for assessing lexical abilities (Gernsbacher and Pripas-Kapit, 2012), the absence of difference on the control conditions (optimal items and logical false items) suggests similar language abilities in both clinical groups with respect to the crucial elements in our study, that is, the understanding of the task instructions and of the terms and items used in the stories. Hence, we acknowledge that verbal abilities might play a role in the dichotomized pattern observed in the ASD group and in the IQ-matched groups, i.e., their abilities with scalar inferences might be commensurate to their language abilities.

At the same time, we also believe that difficulties with scalars might reflect poor level in the other components of intelligence in general, for instance mental flexibility. Mental flexibility is a necessary component to adapt expressions that are on a scale to the context of use. Consistently, children with ASD show particular difficulties in solving ambiguous words, due to their difficulty of use the context to solve ambiguity (Happé and Frith, 2006). Overall, thus, our findings indicate a disruption of pragmatics in ASD, which is linked to individual intellectual abilities, possibly at both verbal and performance level. Further considerations are not licensed by our study, since we did not differentiate between different aspects of the IQ measure. Future studies should aim at disentangling this conundrum and the different weight of cognitive capacities, possibly using finer grained measures of both lexical and non-linguistic skills.

The absence of significant differences between the ASD and the IQ-matched groups in the “I agree a bit”-answers for underinformative statements points to another important aspect. Given the role of general cognitive and verbal abilities, pragmatic difficulties, although tied to ASD, may be not exclusive to ASD. A recent volume lists a large number of developmental disorders and clinical conditions where pragmatic language disorder is observed (Cummings, 2017). Focusing on developmental disorders and on the specific domain of scalars, also children with Specific Language Impairment were shown to perform poorly compared with TD children (Katsos et al., 2011). Interestingly, their difficulty was shown to be proportionate to their language level, in line with our observation that difficulties with underinformative statements experienced by ASD children are proportionate to their IQ (both performance and verbal) level. Pragmatic difficulties are reported also in mental illness, affecting the domains of figurative language and conversation (Bambini et al., 2016a), as well as scalars (Wampers et al., 2017). Also neurological patients are impaired with pragmatics, in figurative language and discourse (Bambini et al., 2016b; Carotenuto et al., 2018), as well as in scalar inferences (Spotorno et al., 2015). All these populations exhibit impairment in other cognitive domains too. We believe that a relevant area of investigation for future research might indeed lie in disentangling the cognitive substrates that might differently underlying pragmatic difficulties across populations, to ascertain the impact of pragmatic language disorder *per se* (i.e., not dependent on other kinds of verbal or cognitive problems) in developmental as well as in acquired conditions.

After discussing the role of intellectual and language abilities, one might question if theory of mind abilities plays a role. Indeed, most of the debate on pragmatics in ASD has been devoted to the role of theory of mind in determining pragmatic behavior (Baron-Cohen, 1988). For instance, for metaphor comprehension, early literature strongly emphasized the role of mind reading skills (Happé, 1993), whereas more recent studies pointed to vocabulary as the best predictor of metaphor understanding (Norbury, 2005; Kalandadze et al., 2018). Conversely, the theory of mind requirement is considered to be higher for other pragmatic phenomena that require reasoning about mental states, for instance irony (Andrés-Roqueta and Katsos, 2017). For scalars, authors have argued that the theory of mind load is low (Pijnacker et al., 2009), since scalar implicatures require first-order mental states (e.g., Marcus does not know that…), but not second-order or higher order states (e.g., Marcus does not know that Philip knows that…). Also, the knowledge required to successfully perform the typical tasks with underinformative statements is visually accessible and shared between the child and the character in the task, therefore posing minimal demand on theory of mind (Andrés-Roqueta and Katsos, 2017). Consistently, studies showed that autistic-like traits are not crucial in the comprehension of scalars (Heyman and Schaeken, 2015; Antoniou et al., 2016). Our results of the IQ-matched group are in line with this idea, suggesting that verbal intelligence and mental flexibility might have a greater role than theory of mind. However, the study by Hochstein et al. (2017) reported that ASD individuals, although performing like controls in the binary evaluation tasks on scalars, differed from controls and may not spontaneously take into account the partner’s epistemic state. Given this evidence, we cannot exclude that reasoning about mental states plays some role in pragmatic tolerance.

As a last point, we shall consider the implications of our results for pragmatic theory and specifically theories on scalars. First, we believe that our data might offer some hints on one of most discussed topics in the theoretical and experimental pragmatics literature on scalars, namely the automatic vs. non-automatic debate (Bott and Noveck, 2004; De Neys and Schaeken, 2007). Our data do not actually offer direct evidence in support of either account. However, the difference between Experiments 1 and 2 suggests that competence with scalar implicatures is not a yes/no alternative, but rather a fine-grained dimension where different levels are possible. The preference for the intermediate answer between acceptance and rejection suggests that scalar inference is actually a matter of degree, which is more in line with the idea that such inference is not derived automatically but rather through a process that might require acceptance of ambiguity and further pragmatic enrichment of meaning. Future theorizing should take into account this aspect of processing, and include different tolerance levels into models.

Second, our data, specifically the difference between scalar and non-scalar items, might speak to the debate over the context dependency of scalar implicature (Katsos, 2009). While, as explained already, there is abundant evidence that children struggle with scalar implicature tasks with the underinformative *some*, there is also evidence that they are somewhat more successful at computing non-scalar or *ad hoc* implicatures, which do not depend on lexical scales but on context (Papafragou and Tantalou, 2004; Stiller et al., 2015; Horowitz et al., 2017). However, there is no consensus. Katsos and Bishop (2011), for instance, observed in both the binary and ternary task that the children’s performance did not significantly differ between scalar and non-scalar expressions in the underinformative condition. For Experiment 1, the regression analysis revealed a significant interaction between Scalar-Type and Informativeness-Type, with unexpectedly more pragmatic responses (“I disagree”) given on scalar underinformative statements compared with non-scalar underinformative statements. Although surprising for children, this result is in line with some results with adults. For instance, in the Katsos and Bishop observed that not all adult responses in the experiment with binary options were a straightforward acceptance or a rejection, but were more indirect, that is, phrased as revisions or meta-linguistic remarks (Katsos and Bishop, 2011). Importantly, adults gave more straightforward categorical rejections (i.e., pragmatic responses) for underinformative utterances with scalars than with non-scalar statements. An explanation for such an effect might be that one can only rely on the context (that is, what one observed) for the non-scalar statements, while the words themselves (e.g., ‘some’) give an additional clue in the case of scalar statements. However, the regression analysis of Experiment 2 showed another picture. It revealed a significant interaction between Group and Scalar-Type. Indeed, we observed more “I agree a bit”-answers with the non-scalar items than with the scalar items for both the underinformative (which is appropriate) and the logical false items (which is inappropriate) for the ASD group and the IQ-matched group compared with the TD group. The most straightforward explanation for the higher number of “I agree a bit”-answers on the underinformative non-scalar items is that the non-scalars or *ad hoc* implicatures are easier and therefore elicit more nuanced pragmatic responses. This observation is clearly more in line with the majority of the literature than the opposite pattern in Experiment 1. The fact that this difference is significant for the ASD group also calls for a qualification of our previous claim that the ASD group is pragmatically intolerant. For the *ad hoc* non-scalar items, one only has to consult the context which offers the relevant alternatives, while, for the scalar items, one has the context but also the lexical meaning to deal with. This might cause an extra processing difficulty, which leads to fewer pragmatic “I agree a bit”-answers. It might be that a more fine-grained measurement is necessary to reveal these differences, which is why the effect is not present in Experiment 1. Of course we have to acknowledge that in Experiment 1, the interaction between Group and Scalar-Type was not only not significant, but was even pointing in the other direction, that is, more logical “I agree”-answers for the ASD group on the underinformative statements. Moreover, the higher number of “I agree a bit” answers on the logical false items in Experiment 2 does not fit very well with the sketched account of a higher difficulty of the scalar items for the ASD group. However, it is possible to reconcile the results of the two experiments if we interpret the “I agree a bit”-answers of the ASD group not so much as a reflection of a higher pragmatic tolerance, but rather as an indication of a higher degree of uncertainty. This would mean that in Experiment 2 the non-scalar underinformative and logically false items turned out to be more difficult than their scalar counterparts, because they elicited more “I agree a bit”-answers. The participants in our study are older than most of the children in the studies mentioned above. It might therefore be, as suggested by Katsos and Bishop (2011), that their exposure to context-independent scales of informativeness was higher, which might have caused the relative ease of the scalar items. It is clear that further research has to clarify this observation and tentative explanation further, both the difference scalar and *ad hoc* non-scalar items in general and the difference between them for the ASD group.

Notwithstanding all reservations specified above, given the observed differences between scalar and non-scalar items, our results are less in line with the unitary account of pragmatic inferencing (Sperber and Wilson, 1995; Carston, 1998; Geurts, 2010), which collapses the distinction between scalar and non-scalar implicatures on the grounds that both rely on contextually specified expectations of informativeness. Although somewhat contradictory, our results of Experiments 1 and 2 are more in line with theorists who claim that scalar and non-scalar implicatures are different. Some of these accounts (Levinson, 2000; Chierchia, 2004) argue that context-independent implicatures are privileged compared with context-dependent implicatures and therefore easier and acquired earlier. The results of Experiment 1 are in line with this claim, whereas the results of Experiment 2 are less. It is clear that the differences between scalar and non-scalar *ad hoc* expressions are not straightforward, and that even subtle factors such as the naturalness of the sets involved can have an influence (Katsos and Cummins, 2012). Again, future experiments with the ternary task might help gaining more conclusive evidence on this debate.

Finally, from the point of view of developmental pragmatics, our findings confirm that TD children are tolerant to underinformativeness and the middle option is preferred over the extreme ones, in line with previous evidence (Katsos and Bishop, 2011). If tolerance can be seen as a step toward a full-fledged mastery of pragmatic competence, the ASD group seems to lag behind in the process of pragmatic acquisition, since ASD children do not show the tolerance typical of the same age TD children. Therefore, the ternary judgment task might prove useful in future studies to draw the developmental trajectories of pragmatic competence in the domain of informativeness.

## Conclusion

Overall, our study adds a piece to the puzzle of the ASD pragmatic profile. In typical conditions, developing a competence with scalar expressions is a complex process that involves also a stage of tolerance to informativeness. The use of a fine-grained ternary task allowed us to see a different tolerance to informativeness in ASD, which might be disguised by the forced option in the classic binary task. Specifically, ASD children tend to either fully agree or fully disagree with underinformative statements, in contrast with the preference for middle options in TD children. A dichotomized attitude toward the speaker’s meaning might hinder dealing with the broad category of implied meanings that arise from adhering to the Maxim of Quantity in communication. Ultimately, thus, pragmatic tolerance might be an area of intervention for improving social communication skills of individuals with ASD.

## Author Contributions

WS and VB designed the study. MVH prepared the experiments, constructed the stimuli, performed the experiments, and did the statistical analysis under supervision of WS. MVH wrote the first draft of the method and result section. WS and VB did the data interpretation, wrote the introduction and the general discussion, and revised the methods and results sections. All authors contributed to this article, both substantively and formally, and approved the final version of the manuscript.

## Conflict of Interest Statement

The authors declare that the research was conducted in the absence of any commercial or financial relationships that could be construed as a potential conflict of interest.
